# Advantages of Graphene Biosensors for Human Stem Cell Therapy Potency Assays

**DOI:** 10.1155/2018/1676851

**Published:** 2018-05-29

**Authors:** Roxana-Maria Amărandi, Diana F. Becheru, George M. Vlăsceanu, Mariana Ioniță, Jorge S. Burns

**Affiliations:** ^1^Faculty of Medical Engineering, University Politehnica of Bucharest, Gh. Polizu 1-7, 011061 Bucharest, Romania; ^2^Advanced Polymer Materials Group, University Politehnica of Bucharest, Gh. Polizu 1-7, 011061 Bucharest, Romania; ^3^Department of Medical and Surgical Sciences of Children and Adults, University Hospital of Modena and Reggio Emilia, Modena, Italy

## Abstract

Regenerative medicine is challenged by the need to conform to rigorous guidelines for establishing safe and effective development and translation of stem cell-based therapies. Counteracting widespread concerns regarding unproven cell therapies, stringent cell-based assays seek not only to avoid harm but also to enhance quality and efficacy. Potency indicates that the cells are functionally fit for purpose before they are administered to the patient. It is a paramount quantitative critical quality attribute serving as a decisive release criterion. Given a broad range of stem cell types and therapeutic contexts the potency assay often comprises one of the most demanding hurdles for release of a cell therapy medicinal product. With need for improved biomarker assessment and expedited measurement, recent advances in graphene-based biosensors suggest that they are poised to be valuable platforms for accelerating potency assay development. Among several potential advantages, they offer versatility for sensitive measurement of a broad range of potential biomarker types, cell biocompatibility for direct measurement, and small sample sufficiency, plus ease of use and point-of-care applicability.

## 1. Introduction

A wide range of novel Advanced Therapy Medical Products (ATMP) have been pursued intensively over the last decade. In addition to gene therapy medical products (GTMP) and tissue-engineered products (TEP), the application of stem cells has driven extensive research into somatic cell therapy medicinal products (CTMPs). Although the number of ATMPs in the centralised European Union (EU) Marketing Authorization (MA) phase has been described as low [1, 2], a number of recent advances in stem cell biology, complementary technologies, and legislation are collaborating to promote market licensing and cell therapy in clinical practice.

With regard to stem cell-based therapies, our growing understanding of one of the most actively investigated cell types, commonly known as human “mesenchymal stem cell” (hMSC), is fostering debate. Arising from studies of nonhematopoietic human bone marrow stromal cells (hBMSC), an authoritative view is that tissue-specific stem/progenitor cells, a subset of which are skeletal stem cells, are not to be confused with similarly named “hMSC” derived from other tissue sources, especially for regeneration of bone or cartilage tissue [3]. Rather, hMSCs as multipotent stem cells for the skeleton and guardians of lifelong bone turnover are not identical to “hMSC” derived from other anatomical sources such as adipose tissue, muscle tissue, or umbilical cord-derived stromal cells [4]. Key to understanding their potential clinical function is appreciation that they are derived from a perivascular niche [5] incorporated as CD146+ adventitial reticular cells. Their clinical mode of action may be other than formation of regenerating tissue-producing cells, instead reflecting secretion of immunomodulatory and trophic factors that modulate host tissue functions [6]. This does not necessarily totally replace data-driven concepts that hMSC can function via a stem cell tissue integrating nature, especially in homologous contexts [7]. Widening the scope of stem cell therapies has improved insights into how normal epithelial stem cells maintain healthy tissues and how they might be subverted in cancer [8]. Moreover, induced pluripotent stem cells (iPSCs) differentiated as a sheet of retinal pigment epithelial cells are also entering the clinical trial arena [9].

Complementing progress in understanding the diversity of stem cells are advances in large-scale production of therapeutic cells, including bioreactor systems for mesenchymal stem cells [10]. Cell expansion ex vivo may be inevitable for sourced cells to reach a critical clinical dose [11], emphasising need for current good manufacturing practices (cGMP) and above all, conditions that optimise safety [12]. The diverse range of CTMP and concern for malpractice from business marketing of unproven stem cell therapy interventions makes the counteractive measure of strict guidelines fundamental [13]. Human cell therapy potency assays play a major role in establishing ethical practice and improved biosensors for cell analysis are likely to be of great service in the potency assay context.

## 2. The Challenge of Potency in Cell-Based Therapeutics

Among key requirements for cell-based therapy for regenerative medicine, current guidelines stipulate identity, safety [14], purity, and potency as critical quality attributes (CQA) of CTMP. For pharmaceuticals, potency can directly link quantity of the active substance and the product's desired therapeutic effect. The picture is less clear for cell-based products, where the definition of potency needs adaptation to fit the specific properties of cell therapies to also include measurements of viability, self-renewal, death, and differentiation [15]. Definitions of potency can be found in the 1999 European Medicines Agency (EMA) ICH Q6B guidelines, as well as the 2011 Guidance for Industry from the US Department of Health and Human Services Food and Drug Administration (FDA) “Potency Tests for Cellular and Gene Therapy Products” (CGT) [16]. The EMA definition, “the measure of the biological activity using a suitably quantitative biological assay (also called potency assay or bioassay), based on the attribute of the product which is linked to the relevant biological properties” is broadly consistent with the FDA 21 CFR Part 600.3(s) stipulation: “the specific ability or capacity of the product, as indicated by appropriate laboratory tests or by adequately controlled clinical data obtained through the administration of the product in the manner intended, to effect a given result.” Notably, potency measures are tailored specifically for a particular product and guidelines recognize need for flexibility regarding specific types of potency assay or the proposed acceptance criteria for product release. Several potency assay formats can be accepted, including the use of direct or indirect (surrogate markers) indicators of intended biological activity, such as gene expression patterns, cell surface markers, or other biological entities relevant to the desired therapeutic effect, particularly if the CTMP is destined for tissue repair and regeneration. Regional jurisdiction guidelines between Japan the USA and EU differ [17]. The latest updated FDA Guidelines (https://www.fda.gov/downloads/BiologicsBloodVaccines/GuidanceComplianceRegulatoryInformation/Guidances/CellularandGeneTherapy/UCM585403.pdf) indicate that to lawfully market a biological product a biologics license must be in effect issued only after determination that the product meets safety, purity, and potency standards.

The challenges to potency assay development are many: (i) inherent heterogeneity in the starting cell population; (ii) a limited cell product lot size and amount available for testing; (iii) limited viability and stability of cellular products; (iv) the difficulties in establishing the mechanism of action (MOA) considering the numerous intrinsic factors and active components; (v) the potential for both positive or negative interactions among active components; (vi) the difficulty of establishing reference standards; (vii) additional complexity if biomaterials are involved; (viii) the difficulty of accurately predicting the in vivo fate from external measurements made before cell administration to the patient.

**Figure 1:**
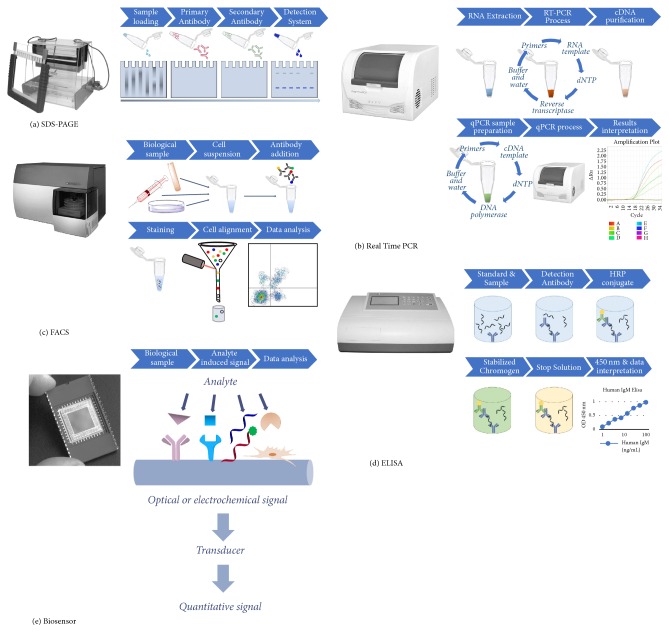
Biosensors for potency assay simplification. Traditional potency assay measurement technologies include (a) Protein expression via Sodium Dodecyl Sulphate-PolyAcrylamide Gel Electrophoresis (SDS-PAGE), (b) Gene expression via quantitative Real Time Polymerase Chain Reaction (qRT-PCR), (c) Live cell flow cytometry via Fluorescence-Activated Cell Sorting (FACS) analysis, or (d) Enzyme-Linked Immunosorbent Assay (ELISA) for antibody targets. These are typically multistep procedures requiring more time and expertise than needed for application of (e) dedicated biosensors tailored for specific target analytes.

For the widely used hBMSC, starting material heterogeneity concerns not only identity and parity with minimal defining criteria [18], but also the mode of manufacture [19]. Moreover, for the potency assay, variability also extends to achieving conditions that allow proper phenotypic expression of receptors and active component molecules. The potency assay may be performed in animals and for some measurements this remains necessary; however, this introduces considerable costs for specialized facilities and expertise. In particular, data acquisition and processing need to be confined to time frames compatible with cell harvest, expansion, and administration that prevalently involves use of freshly grown cells, until cryopreservation of stem cells becomes more widely established [20–22]. Assuming the arduous investigation of mechanism of action has reduced complexity to definitive biomarkers, there remain concerns that the method used to make the measurement must be trustworthy and in particular, accurate, sensitive, specific, precise, and robust. Precision relates to measurement reliability and reproducibility, yet robustness is needed for the assay to remain consistent when applied in different clinical sites. Traditional assay methods requiring multistep procedures and operator intervention are likely to be less robust that integrative biosensor devices (Figure 1). Additional concerns are whether handling procedures interfere with potency assays [23] and whether minimising time between cell potency measurement and point-of-care (POC) use improves quality consistency.

Notably, clinical study data is not of practical use for establishing the potency assay. The potency assay predicts ability to cause functional effect rather than clinical effectiveness or outcome and needs to be capable of defining individual product lot release criteria. The emerging apparent contradiction is that potency assays benefit from being highly sophisticated yet technically simple. To date, many potency assays rely on definitive end points that match relatively clear phenotypes such as cell proliferation, differentiation, cell death, and ability to induce angiogenesis. Not all traditional assays measuring these phenotypes are necessarily equivalent however, especially for diverse therapeutic cell types and indications. Whereas proliferation may be a phenotype considered useful for evaluating the potency of autologous hematopoietic stem cells for bone marrow transplantation, interlaboratory reproducibility of commonly performed colony forming unit (CFU) assays is problematical [24]. Sample tests may poorly reflect the administered bulk clinical product and closely monitoring prompt engraftment after administration remains one of the most reliable indicators of quality [25]. For this and umbilical cord blood potency assessment [26], rapid functional assays are required. Derivation of a potency assay includes identification, qualification, and maintenance of a product-specific reference standard for direct comparison in the quantitative potency assay, to obtain a potency ratio used to define product release criteria [16].

**Figure 2:**
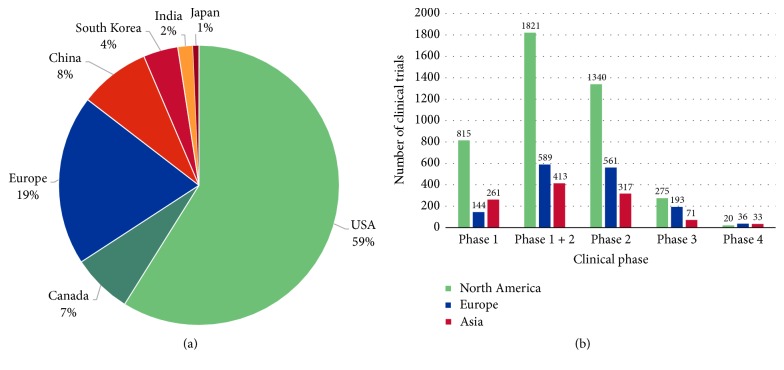
Graphic representation of the relative number of stem cell clinical trials. (a) According to geographical region, (b) number of stem cells studies for each particular clinical phase.

Understandably, the high costs and investigative time needed for developing a suitable potency assay for cell-based products has hindered entry of biologicals into phase III or IV clinical trials. A potency assay needs to be one of the first considerations for any CTMP, yet there is currently a marked discrepancy between the number of phase I/II clinical safety trials and phase III or IV clinical trials that require a validated potency assay (Table 1). For example, out of all the stem cell-related clinical trials registered on https://clinicaltrials.gov for the US, only 8.58% are in phase III or IV. For China this index is closer to 20% and in EU and Japan 30%; but figures also reflect relatively fewer early stage stem cell clinical trial studies outside the US (Table 1, Figure 2). High installation and implementation costs of cell analysis systems and stringent approval regulations may delay growth of CTMP.

## 3. Current Potency Assays in Advanced Therapy Medical Products

The first stem cell-based ATMP therapy granted marketing authorization by EMA, Holoclar, treats severe trauma induced limbal stem cell deficiency (LSCD) in adults [27]. A recommended dose of 79,000 to 316,000 cells/cm^2^ can suffice to cover the entire corneal surface of the patient's affected eye. Corneal epithelium that would otherwise be irrevocably damaged is replaced with epithelium with a reservoir of continually regenerating limbal stem cells that provide long-term normal corneal function. Marketing approval came eighteen years after initial proof-of-principle success in two patients [28] and a 2010 clinical study that showed permanent restoration of a transparent renewing corneal epithelium in 76.6% of 112 patients [29]. Product potency assay development followed the observation that p63^bright^ expression in the stem cell nuclei of holoclones could be linked to a good clinical outcome. Providing a potency reference, it was found that among the total number of clonogenic cells, presence of >3% holoclone-forming limbal stem cells correlated with successful transplantation [29]. Thus, quantification of p63^bright^ cells could serve as a key potency assay biomarker for this cell-based therapy.

Several other stem cell CTMP have resorted to quantitative ELISA methods in potency assays. Multistem®; an adult allogeneic bone marrow-derived product has shown beneficial effects in animal models of ischemic injury. For potency assays regarding complex indications, when an authentic bioassay is not feasible, surrogate in vitro assays identify biological activity analytically by correlation to a relevant product-specific causal activity. Angiogenic factors secreted by Multistem multipotent adult progenitor cell (MAPC) populations, measured by enzyme-linked immunosorbent assay (ELISA) correlated with induction of tube formation by endothelial cells in vitro for a proposed quantitative potency assay with predefined accept/reject criteria [30]. A more sophisticated aortic ring potency assay could quantitatively evaluate the ability and potency of cellular therapy candidates to migrate to areas of angiogenesis, influence ECM processing, and contribute to vessel development via physical contact [31]. The angiogenic activity of a pooled ex vivo expanded allogeneic human bone marrow mesenchymal stromal cell product Stempeucel® identified vascular endothelial growth factor (VEGF) measured by ELISA as a dose-dependent active effector. A VEGF concentration of at least 2 ng/ml/million hMSCs was estimated to be sufficient for the cell therapy product to induce blood vessel formation [32]. It remains important to corroborate such ex vivo measurements with correlation for expected specific biological response in vivo. The cells may secrete additional factors such as glycine [33] that might also govern in vivo outcome [34].

An effective surrogate potency measure for the immunoregulatory activity of the Osiris Bone marrow adult mesenchymal stem cell product Prochymal™ was an ELISA measure of tumor necrosis factor receptor 1 (TNFR1) levels in the cell therapy product, in combination with a qualitative measurement of the inhibition of the interleukin 2 receptor *α* (IL2R*α*) expression on activated T cells. Quantitative metrics included a concentration of at least 13 pg TNFR1 per million MSCs and the ability of inhibiting at least 30% of IL2R*α* expression in cocultured CD3/CD28-activated peripheral blood mononuclear cells (PBMCs) relative to control was considered sufficient in order to induce the desired therapeutic effect [35].

The product NurOwn® consists of autologous ex vivo-propagated bone marrow-derived MSCs induced to secrete neurotrophic factors (MSC-NTF cells), currently undergoing a phase III clinical trial for the treatment of Amyotrophic Lateral Sclerosis (ALS). Biomarkers quantified for this stem cell-based therapy product include cell-secreted neurotrophic factors, inflammatory factors, and cytokines in the cerebrospinal fluid (NCT03280056). Notably, microRNA profiling of MSC-NTF cells could distinguish them from matched origin MSC, characterisation that could be useful for a potency assay if biological response is dependent on a threshold number of MSC-NTF cells [36].

The NiCord® product consists of a cryopreserved stem cell product consisting of allogeneic ex vivo-expanded umbilical cord-derived hematopoietic CD34+ progenitor cells and the noncultured cell fraction of the same cord blood unit. Current phase 3 clinical trials include treatment of hematological malignancies such as acute lymphoid leukemia or myelodysplastic syndrome (NCT02730299). An effective measurement of biological effect for this product was the time for neutrophil engraftment following transplantation [37]; however, this has yet to strictly conform to the potency assay requisite of measurement before administration.

Numerous other stem cell-containing cell-based therapy products are currently pending market approval or are undergoing clinical trial evaluation. Notably, the respective definitive potency assays are either proprietary or still a work in progress. Nonetheless, they serve as good examples for considering the scope of applications where graphene-based biosensors might be helpful in early stages of cell therapy development. The blood and tissue bank of Catalonia product XCEL-MT-OSTEO-ALPHA, representing autologous ex vivo-expanded MSCs fixed in allogeneic bone tissue, is currently being tested in phase 1/2 clinical trials for the treatment of spinal fusion (NCT01552707), hypertrophic pseudoarthrosis of long bones (NCT02230514) and femoral head osteonecrosis (NCT01605383). The product PneumoStem® consists of allogeneic ex vivo-expanded human umbilical cord blood-derived MSCs currently in phase 1/2 clinical trials for the prevention of bronchopulmonary dysplasia in premature infants (NCT02381366), as well as a phase II clinical trial for the treatment of intraventricular hemorrhage (NCT02890953). The results of these trials will be important to evaluate the safety and effectiveness of this approach [38]. CordIn™ consists of allogeneic ex vivo-expanded umbilical cord blood-derived CD133+ cells, currently in phase 1/2 clinical trials for the treatment of sickle cell disease and thalassemia (NCT02504619) plus severe aplastic anemia and myelodysplastic syndrome (NCT03173937). The product NeuroStem® consisting of allogeneic human umbilical cord-derived MSCs is undergoing phase 1/2 clinical trials for the treatment of Alzheimer's disease (NCT02054208). The product CartiStem®, allogeneic ex vivo-expanded umbilical cord blood-derived MSCs in combination with sodium hyaluronate, is under evaluation in two parallel clinical trials for the treatment of knee chondral defects (NCT01733186) and osteoarthritis (NCT01041001). Although a particular potency assay was not described and biological activity including inhibition of proinflammatory cytokines is poorly characterised, long-term benefit has been reported [39].

## 4. Graphene-Based Platforms to Accelerate Potency Assay Development

Currently, CTMP research is well-poised for collaborative development of dedicated biosensors for monitoring cell potency, adopting principles of quality by design (QbD) for cell product manufacture [40]. Existing commercial cell-based products already provide qualifying bioactive mediators as target molecules or analytes that can help guide potency biosensor design strategies.

Although ELISA and PCR-based quantification seem to be the methods of choice in the current development of stem cell-based products due to their outstanding detection limits, the challenge of guaranteeing high quality measurements is increasingly being met by progress in biosensor design. Various functionalized forms of graphene and nanocomposites [41] can be used to develop biosensors for a broad range of probes suitable for recognizing specific biomarkers relevant to stem cell-based therapies, including hallmarks of vascularization (e.g., VEGF [42]), proliferation and differentiation (e.g., miR-21 [43] and Bcl-2 [44]), immunity (e.g., TNF-*α* [45] and IFN-*γ* [46]) or apoptosis (e.g., caspase-3 activity [47]), and pluripotency factors (e.g., NANOG [48]). Graphene-based materials include pristine graphene, functionalized forms such as graphene oxide (GO), reduced graphene oxide (rGO), and graphene quantum dots (GQD) can each introduce particular charge interaction qualities utilized by sensing platforms [49].

**Figure 3:**
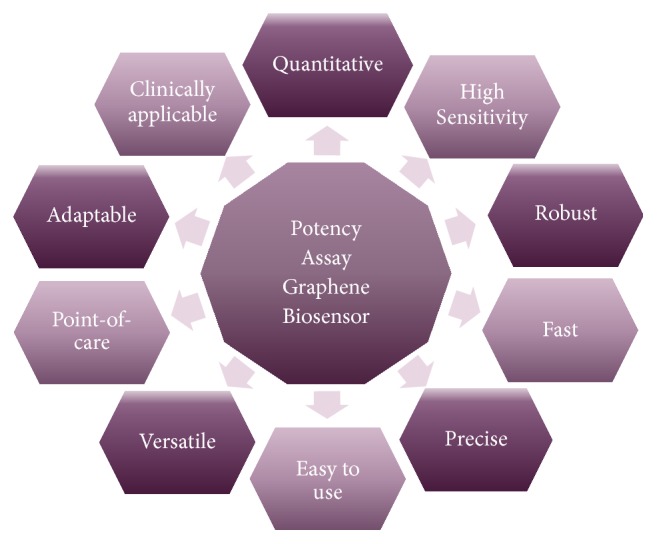
Advantages of graphene biosensors for potency assays.

This versatility can help advance development of novel potency assays for future stem cell-based products (Figure 3). Graphene-based biosensor platforms allow biocompatibility and relatively straightforward applicability to cell product fabrication procedures, scalability for small sized samples, and, above all, an opportunity to create innovative enhanced cell interactive microenvironments [50] tuned to optimal measurement.

Stem cells are characterised by a capacity for both self-renewal and asymmetric division that produces one identical daughter stem cell and a second distinct daughter cell equipped with the potential to commit to a lineage-specific differentiation program [51]. Found in virtually all tissues of the body [52], determining which stem cells are the most potent is far from resolved [53], but it is clear that, without a universal stem cell type or method of delivery, long-recognized characterisation challenges remain [54]. Nonetheless, there is growing consensus that changes in DNA-binding core histones regulate cell lineage commitment [55] and may help characterise self-renewing stem cells [56]. A valid concern is that pretreatment in vitro manipulation of the therapeutic cells can impair their subsequent biological performance [35, 57]. In this regard, biomarkers indicating cell stress responses can guide proper manipulation of such cells for their therapeutic context and enhance clinical outcomes [36, 58].

Consistent with providing improved conditions for monitoring stem cell performance, 3D GO-encapsulated gold nanoparticles could serve as nondestructive biosensors of neural stem cell (NSC) differentiation potential. An intrinsic property of graphene is enhanced adherence to molecules that contain aromatic structures. Highly unsaturated metabolites are predominant in undifferentiated stem cells so that Raman spectroscopy peaks of undifferentiated NSC on GO-encapsulated gold nanoparticles were 3.5 times higher than peaks obtained from control metal structures and clearly distinguishable from peaks obtained using differentiated cells that oxidize the metabolites upon differentiation [59]. Given that such metabolic changes characterise differentiation in other stem cell types, this nondestructive in situ monitoring tool may have broad applicability.

Nanomaterials may be particularly useful in enabling more specific measurements in vivo and this may help bridge the in vitro/in vivo divide, so that what is measured in vitro during cell expansion is genuinely more relevant for the desired therapeutic approach. Graphene oxide can be incorporated in a number of nanocomposites to serve as a platform that enhances electrical properties for biosensing applications in vivo [60]. The unique GO quality of bearing hydrophilic groups on its basal surface enhances the diversity of possible molecule conjugations for functionalization and the raised water affinity allows its integration in 3D scaffold hydrogels for the practical concept of injectable biosensors responsive to long wavelength light [61]. Thus, graphene-based sensing in vivo may be used to establish best stem cell implantation conditions, plus suitable dose and application time frames, and parameters of key significance in potency assay development.

As highlighted by the examples above, among the enhanced healing qualities required from cell-based therapies [62], the following are frequently in demand: (i) resisting apoptosis and blocking extensive cell death that accompanies tissue injury [63]; (ii) the ability to promote angiogenesis with integration of the host circulatory system [31]; (iii) induction of tissue regeneration that may include stimulation of local progenitor cells or site-specific integration and differentiation [34, 64]; (iv) modulation of the innate or adaptive immune system, for example, to enhance transplant engraftment by reducing the recipient immune response or attenuate donor tissue recognizing the recipient host as foreign in graft versus host disease (GvHD) patients [65].

For a definitive assay, cell death is a great endpoint. Governing the process of apoptosis or programmed cell death, a cascade of molecular events usually activates a cysteine-dependent aspartate directed protease (caspases), an enzyme family with multiple roles in regulating stem cell properties [66]. GO could enhance electrochemical signal amplification to derive a very sensitive caspase-3 sensor with a low detection limit of 0.06 pg mL^−1^ [67]. Two proteins often used to monitor apoptosis in tumor cells, the crucial regulators B-cell lymphoma 2 (Bcl-2), and Bcl-2 associated X protein (Bax) can prevent or enhance apoptosis, respectively. Their detection can be used to estimate the suitable dose of a cell death inducing therapy. Incorporating rGO on a glassy carbon electrode (GCE) can increase the surface area and provide a substrate for immobilizing specific antibodies. Combined with nanoparticles, an electrochemical biosensor was developed with detection of apoptosis regulators achieved using as few as 1000 cells [44].

Demonstrating the versatile manner by which graphene platforms can be used to detect potency assay-relevant molecules, a number of different biosensor types have been developed for the angiogenic growth factor VEGF. A field-effect transistor (FET) electronic platform conjugated with VEGF-specific RNA aptamers could recognize target molecules at an unprecedented 100 fM concentration [68]. Alternatively, for an optical biosensor design, graphene was employed as a superquencher to reduce background signal levels. An amplified fluorescence aptasensor beacon and nicking enzyme platform showed high VEGF sensitivity and selectivity [69]. Moreover, a reusable biosensor was developed using a magnetic GO-modified Au electrode that could detect VEGF in complex fluids such as human plasma [70].

Stem cell integration in tissues was not only critical for successful corneal recovery using limbal epithelial stem cells but was also found to be important for mesenchymal stem cells in cementogenesis, a process establishing regeneration of cementum for anchoring teeth to alveolar bone. Sorting periodontal ligament cells according to CD146 expression could homogenize cultures and enrich cells with high colony forming potential, capable of subsequently resurfacing dentin with a newly formed cementum-like layer, allowing improved integration in the dentin surface [68]. Modifying glassy carbon electrodes with reduced graphene oxide-tetraethylene pentamine (rGO-TEPA) served as a platform for a secondary antibody targeting TiO2 nanospheres detecting CD146 antigen. The ultrasensitive immunosensor achieved a wide linear range (0.0050–20 ng mL^−1^, with a low detection limit of 1.6 pg mL^−1^, and good reproducibility and stability, qualities that are key for good potency assays.

Immunomodulatory mechanisms achieved by mesenchymal stem cells remain to be investigated [71] and it is appreciated that identification of functional markers of potency with easily applicable methods of measurement would be of benefit to the field [72]. It is increasingly appreciated that hMSC can secrete biologically active extracellular vesicles (EV) including exosomes and microvesicles (MV) that can mediate cell-to-cell communication and cell signalling [73]. Recently, an in vitro immunomodulation potency assay was devised to reproducibly measure the dose-dependent inhibitory effect of hMSC-derived EV on induced T-cell proliferation [74]. Further development of the potency assay could exploit a new microfluidic exosome analysis platform based on a novel graphene oxide/polydopamine (GO/PDA) nanointerface that greatly improved exosome immunocapture whilst suppressing nonspecific exosome adsorption. With a 4-log dynamic range, EV analysis could be performed on just 2 *μ*L of plasma without sample processing [75].

## 5. Stem Cell Peculiarity and Fit for Purpose Graphene-Based Biosensors

Bone repair is an intensively explored regenerative application for the most commonly employed hBMSC cell type under investigation in clinical trials. Cellular products in the form of allografts containing mesenchymal stem that are currently evaluated for safety and efficacy in many phase I/II clinical trials will require suitable potency assays for progression to subsequent clinical trial phases. Enhanced osteogenesis serves as a prime example where graphene-based biosensors present favourable qualities for improving potency assay development. New quantifiable gene expression biomarker candidates are emerging [76, 77] with exploration extending to microRNA regulators of bone regeneration [78]. Sensitive GO based biosensors for quantifying mRNA and microRNA [79, 80] have been described, with low noise and excellent discrimination and agreement with results obtained using qRT-PCR.

An important aspect for any potency assay is that it should fit cell expansion timelines and thus fast analysis is advantageous. The potency assays proposed by Murgia et al. [76] involved verifying an appropriate biological response to an osteogenic induction medium including bone maturation protein (BMP-2). When adopting cGMP culture conditions supplemented with platelet lysate rather than fetal bovine serum (FBS), osteogenic differentiation was accelerated in a manner consistent with earlier observations [81]. This allowed significant gene expression changes to be measured by qRT-PCR within one rather than two weeks. Early measurement at one week was critical, the potency interrelationship was lost for measurements at two weeks. Notably, correlation between gene expression and subsequent bone formation in vivo only worked for a cohort of just five out of twelve tested osteogenic biomarkers. Monolayer culture conditions in vitro provide very limited mimicry of the in vivo microenvironment. Beyond cell-innate universal responses governing initial stem cell differentiation to osteogenic progenitor cells, in vitro conditions do not maintain contextual congruity. So, over time, post-induction cultured cell gene expression patterns will increasingly reflect culture-specific values diverging from in vivo relevance. The selection of particular subsets of early-responder genes would provide a more globally applicable measure, reflecting stem cell to progenitor cell conversion per se, rather than contextual influence. Consistent with this view, some of the potency biomarkers were correlated to molecular changes during bone formation using immortalized hBMSC under alternative in vitro osteogenic induction conditions [82]. Similar to platelet lysate, the effect of GO on hBMSC in vitro was that it positively enhanced osteogenic differentiation [83]. Moreover, graphene substrates could stimulate osteogenic differentiation in bone marrow-derived hBMSC at the same rate observed for cells receiving BMP-2 treatment [84]. The ability of stem cells to be highly responsive to microenvironments implies focusing not only on materials, but also on finely tuned geometries and structures that may be particularly suited for obtaining reliable measurement of the potency assay target. Topographic modification that increased roughness allowed graphene to provide a chemical-free route to inducing differentiation [85]. Minimising the number of constituents required to perform a potency assay will improve its robustness. The observation that GO could enhance osteogenesis in vivo suggested that the in vitro induction of osteogenic differentiation was linked to appropriately relevant biological properties [86–88].

## 6. Future Directions for Graphene-Based Biosensors for Potency Assays

Depending on the nature of the envisioned detection platform, integrating graphene with sustainable production methods will be important for obtaining highly reproducible molecular interactions with biological molecules [89]. Various methods are under development to improve electrochemical, optical, or hybrid signal biosensor platforms including incorporation of graphene in inks for screen-printed graphene electrodes [90]. Nanoscale carbonaceous materials augment two key synergistic factors, a quantum effect and a surface effect and although significant progress remains [91], 3D printing technology introduces new levels of tissue engineering refinement into the patient treatment plan [92]. Excellent prospects for improved mimicry of structural and functional properties of complex tissue and organs are likely to help provide potency assays that are highly specific, with improved measurement within tissue damage contexts [93]. The extent of precision rendered possible by 3D technology makes it more suited for high-tech manufacturing process to accomplish reproducible and customizable multicomponent constructs with precise geometries. Enhancing cartilage tissue engineering, 3D printing technology has been shown to effectively deliver stem cells [94] and GO could induce protective biological signal pathways [95]. Nanomaterial science is expected to enhance the performance of 3D printed devices to unprecedented standards. The chemistry of the inks influences biological signal transduction and that suitable for biosensing needs to meet criteria of biocompatibility, specific affinity, and a processing flow matching a particular viscosity range [96].

Graphenic species are being tested to achieve market grade novel inks for 3D printing applications [97] and means of obtaining more hydrophilic graphene [98]. The printing approach is compatible with feasible biodevice manufacturing [49]. Parameters of printing deposition for graphenic formulations that retain optical and electronical qualities [99] and the possibility of depositing ultralow friction graphene flakes [100] remain compatible with standards for of cell laden bioinks and 3D printing techniques [101]. Incorporation of compatible nanocomposite materials such as chitosan provides an ink platform suitable for the development of modular biosensors [102]. Technological progress for sensors allowing single use [103] or label-free measurement [104] combined with use of sustainable platforms such as paper [105] renders point-of-care use concepts feasible. Nonetheless, considerable multidisciplinary research is required to advance proofs of principle to cell potency assay grade products.

## 7. Conclusions

The burgeoning number of stem cell clinical trials requiring a potency assay provides an excellent opportunity for advanced biosensor design to address valuable clinical applications pragmatically within a preclinical context. The versatility of graphene-based biosensors is well suited for the complex nature of potency assays that will be very specific for each therapeutic application. Different versions of graphene allow prospects for a choice of sensor types, especially given successful fabrication of nanoparticle composites allowing the electrochemical characteristics of graphene to enhance specialized sensing platforms. Graphene oxide presents advantages for water dispersion, biocompatibility, and versatile surface modification and electrochemical transduction of the signal allows miniaturization. This can help establish assays suited to limited cell product lot sizes and facilitate measurement prior to administration with point-of-care tools. Particular advantages for potency assays are likely to be derived from appreciating that stem cells are highly responsive to microenvironments; thus surrogate assays based on closer mimicry of the therapeutic situation are likely to provide better correlative measurement of potency biomarkers [106]. Graphene oxide chemically exhibits an assortment of subtypes, that distinctively interact with relevant biological targets such as DNA, micro-RNA, or mRNA. This improves options for measuring key molecular components governing the MOA of the cellular product. To this end, the nanoscale quality of graphene-based biosensors allowing their integration into 3D constructs may enhance potency biomarker measurement for better simulation of in vivo conditions. Hence, the pursuit for standardization of graphene-based potency assays is complex, comprising versatile options to tailor graphene layer size distribution, morphology, aggregation, and functionalization [41]. Although many issues remain to be resolved, including reproducibility of tuned graphene quality to meet the more stringent demands of a potency bioassay, the current rate of progress is graphene biosensor design and manufacturing methods favour an optimistic outlook. 3D hydrogels and graphene materials can be combined to develop highly sensitive biosensors that enhance scope for more versatile 3D surrogate potency assays. Introducing tunable material properties of degradability and stiffness could directly influence neural progenitor cell behaviour [106] and influence adipose derived stem cell chondrogenic differentiation [107]. A pH-responsive nanocarrier based on modified graphene oxide to promote acid-triggered intracellular release of a soluble drug illustrates the sophistication that can be envisaged, allowing triggered cell-response potency assays [108]. It is likely that more elaborate tissue-engineered biosensors may become useful in biomarker discovery as functional aspects of stem cells may be dependent on 3D derived signals that are not obtained in monolayer cultures. Thus, with numerous desirable aspects for ATMP potency assays, including ease of use, high sensitivity, versatility, and point-of-care applicability, graphene-based biosensors are likely to offer an attractive solution.

## Figures and Tables

**Table 1:** Number of clinical trials with the term “stem cell” in major geographical regions. Data taken from https://clinicaltrials.gov/ for North America and Asia and https://www.clinicaltrialsregister.eu/ctr-search/search for Europe, at 14.12.2017. ^*∗*^Number presented includes clinical studies with phase or status not declared. ^†^The numbers include also studies declared as terminated or prematurely ended.

Country/region	USA	Canada	Europe	China	Republic of Korea	Japan	India
Stem cell clinical trials up to 2018^*∗*^	2360	277	788	326	160	27	70

Phase 1	766	49	144	153	50	11	47
Phase 1 + 2	1671	150	589	228	107	16	62
Phase 2	1211	129	561	183	82	8	44
Phase 3	184	91	193	39	17	8	7
Phase 4	18	2	36	24	9	0	0

Ongoing	908	117	574	141	54	11	12
Suspended	18	2	28	2	0	0	1
Terminated^†^	255	22	108	2	9	1	4
Completed	1020	118	231	48	59	12	27

With results	418	51	107	4	6	6	2
Without results	1942	226	678	322	154	21	68
